# Introducing a new specifier for functional somatic disorder: a psychodynamic approach to investigating emotional factors

**DOI:** 10.3389/fpsyt.2025.1481405

**Published:** 2025-05-06

**Authors:** Daniel Maroti

**Affiliations:** Department of Psychology, Stockholm University, Stockholm, Sweden

**Keywords:** somatic symptom disorder, bodily distress disorder, functional somatic disorder, emotional awareness and expression therapy, intensive short-term dynamic psychotherapy, psychodynamic therapy

## Abstract

Functional Somatic Disorders (FSD) present a significant challenge in the health-care system, characterized by persistent, distressing physical symptoms without sufficient medical or psychiatric explanations. This conceptual analysis explores the psychodynamic approach to understanding emotional factors influencing FSD, proposing a new psychological specifier. While current diagnostic frameworks, such as DSM-5’s Somatic Symptom Disorder (SSD) and ICD-11’s Bodily Distress Disorder (BDD), incorporate psychological components, they do not fully address emotional dynamics. This paper advocates for integrating emotional factors into diagnostic criteria. The proposed specifier focuses on emotional factors such as unresolved grief, trauma, and unmet needs, which can exacerbate or cause somatic symptoms. Six signs indicative of emotional influence on somatic symptoms are discussed, emphasizing a collaborative investigative approach. Incorporating this specifier could enhance diagnostic accuracy, treatment planning, and patient outcomes by acknowledging the interplay between emotional and physical health.

## 1 Introduction

Patients with persistent physical symptoms have been described using various terminologies over the years. Historically, these conditions were labeled as hysteria, psychogenic disorders, psychosomatic illnesses, and somatoform disorders, although these terms have fallen out of favor (1). Currently, a variety of terms are used to describe these symptoms, such as Functional Somatic Syndromes (2), Central Sensitivity Syndromes (3) and Persistent Physical syndromes (4) each with overlapping and unique features. Moreover, the Diagnostic and Statistical Manual of Mental Disorders, Fifth Edition (DSM-5), uses the term Somatic Symptom Disorder (SSD) (5), while the International Classification of Diseases, Eleventh Revision (ICD-11), refers to Bodily Distress Disorder (BDD) (6).

In an effort to standardize the terminology and enhance both research and clinical practice, the EURONET-SOMA group has advocated for the term “Functional Somatic Disorder” (FSD) (7). FSD encompasses patients with distressing physical symptoms lasting more than three months that cannot be explained by any other medical or psychiatric condition (see **Table 1**). Importantly, FSD is not believed to be a disorder of exclusion, instead it is characterized by altered bodily functioning and the disorder manifests as physical symptoms. These symptoms can vary in severity and may include isolated issues such as dizziness or more severe manifestations like hemiplegia, provided there is no organic cause. FSD can present as single-system conditions which does not need to equate but can present like irritable bowel syndrome (IBS) or multi-system issues such as co-occurring IBS and fibromyalgia, often severely impacting daily life and frequently accompanied by psychiatric comorbidities (8).

**Table 1:** Characteristics of functional somatic disorder with the new proposed psychological specifier.

1 **Persistent Bodily Symptoms:** Symptoms should be present for at least three months, though often longer.
2 **Exclusion of Other Conditions:** Somatic symptoms cannot be better explained by other medical diseases or psychiatric conditions.
3 **Psychological Specifier for FSD:** Somatic symptoms co-varies with emotional factors.

The EURONET-SOMA group suggests adding a psychological specifier to the FSD diagnosis to address psychological factors that contribute to symptom persistence beyond the distress caused by the symptoms themselves (7). This specifier would go beyond the criteria for SSD in DSM-5 or BDD in ICD-11, which main focus is on excessive preoccupation and anxiety regarding symptoms. Examples of additional proposed psychological specifiers suggested by The EURONET-SOMA group include a general sense of bodily weakness, specific attributional styles, negative affectivity, and dissatisfaction with previous healthcare.

Despite the inclusion of psychological factors in SSD, BDD and FSD criteria, current frameworks do not fully capture essential psychological elements from a psychodynamic perspective. This article explores the importance of emotional factors in diagnosing and treating FSD, proposing that these factors be considered as part of the diagnostic criteria for SSD and BDD or as a new psychological specifier for FSD.

This paper will first introduce affect-focused psychodynamic treatments and their rational for FSD. Using a collaborative approach, I will then explain six important signs or questions for healthcare professionals to consider when evaluating whether somatic symptoms co-vary with emotional factors. Lastly, I will discuss the implications and limitations of this approach, while still advocating for the consideration of emotional factors as part of the diagnostic criteria for SSD and BDD, or as a new psychological specifier for FSD. In this conceptual analysis, the terms “bodily”, “physical” and “somatic” will be used interchangeably.

## 2 Affect-focused psychodynamic treatments and their rationale for FSD

Affect-focused psychodynamically informed treatments, such as Emotional Awareness and Expression Therapy (EAET) and Intensive Short-Term Dynamic Psychotherapy (ISTDP), have shown significant effectiveness in treating various Functional Somatic Disorders (FSD), resulting in long-lasting reductions in somatic symptoms (9, 10). For instance, EAET has been compared with Cognitive Behavioral Therapy (CBT) in three randomized controlled trials involving patients with fibromyalgia, musculoskeletal pain, or a diverse sample of chronic pain sufferers (11–13). These studies demonstrated that a significantly higher percentage of patients experienced substantial pain reduction with EAET compared to CBT. Across these trials, an average of 30% of patients experienced a reduction in their pain by 50% or more after the group EAET, but only an average of 5.5% in the CBT group. Consequently, psychodynamically informed treatments have been recommended as evidence-based interventions (14).

Affect-focused, psychodynamically informed treatments suggest that, for some individuals with FSD, unresolved emotional distress—such as grief, fear, rage, guilt, or emotional pain—can contribute to or even generate physical symptoms (15). This is supported by findings that patients FSD often struggle with recognizing, expressing, and regulating emotions (16). When emotional experiences remain unprocessed, often due to adverse childhood events, trauma, or victimization, they may alter neural circuits involved in pain and emotion regulation, thereby leading to or exacerbating somatic symptoms. Neuroscientific evidence supports this interplay between emotion and bodily distress. Experimental findings suggest that implicit or unconscious negative emotions can amplify the perception of pain unpleasantness (17), and studies indicate that emotional and physical pain share overlapping neuroanatomical pathways (18). In chronic pain conditions, there is a shift in neural processing from predominantly somatosensory regions to a more complex interaction involving affective and cognitive circuits, reinforcing the idea that pain is not solely a sensory experience but also an emotional and cognitive one (19). Affect-focused psychodynamically informed treatments - especially EAET - is grounded in these conceptual frameworks and leverages neuroplasticity to promote symptom relief. By addressing the emotional underpinnings of pain and fostering emotional processing, EAET helps recalibrate the brain’s threat response system, ultimately reducing pain and other bodily symptoms (20). Mediation analyses support this mechanism, showing that increased emotional processing of stressful life events is associated with a reduction in somatic symptoms (21), even when controlling for depressive symptoms as a competing mediator (22). These findings highlight the importance of targeting emotional awareness and expression as a pathway for symptom relief in patients with FSD.

If emotional factors indeed exacerbate or cause physical symptoms, it is essential to investigate and explore this connection comprehensively. Observations of temporal correlations in a patient’s history can provide initial insights, but the connection is particularly evident through what is termed “emotional palpation” in psychodynamic theory (23). For example, if a patient reports persistent chest pain without cardiac disease activity and experiences a reduction or disappearance of pain upon focusing on feelings of sadness, this strongly suggests an emotional influence on the physical symptoms. To thoroughly investigate this, a collaborative approach is essential.

## 3 Collaborative investigation of emotional factors and somatic symptoms

How can an investigator assess whether physical symptoms co-vary with emotional factors? Adopting a research-like approach is beneficial: Start with a null hypothesis, assuming that physical and emotional factors are unrelated. If this hypothesis can be rejected, it supports the notion of a connection. Repeatedly rejecting the null hypothesis strengthens the evidence for a link between physical and emotional factors. Although this approach may sound technical, it is fundamentally about maintaining an open mind and exploring this connection collaboratively with the patient (see **Table 2**).

**Table 2:** Investigator’s stance in exploring the possible connection between emotional factors and somatic symptoms.

1. **Ask for Permission to Explore:** Specifically inquire whether the patient wants to investigate if emotional factors influence or cause their physical symptoms. Without consent for joint exploration, further investigation cannot proceed.
2. **Adopt a Researcher’s Mindset:** Approach the investigation with the assumption of not knowing the cause of physical symptoms and maintain a null hypothesis regarding the relationship between emotional and physical symptoms, until you can reject it.
3. **Collaborate:** A collaborative approach is crucial for the patient to realize or experience any potential connection between emotional factors and physical symptoms.

The collaborative nature of this process is crucial (24). Some patients with FSD are distrustful of psychological assessments, making it essential to take their complaints seriously while paying attention to cues of emotional activation (25). Therefore, initially, the patient must be asked if they are interested in exploring whether emotional factors are related to or influence their physical symptoms. Without consent, further investigation is not feasible, and the patient should be referred for additional medical care. If the patient is uncertain, further discussion is necessary to obtain a clear answer. Once the patient agrees, the investigation into the possible link between emotional and physical symptoms can proceed collaboratively. Practically, this involves searching for evidence both supporting and opposing the existence of an association between emotional factors and physical symptoms. While no single sign can definitively confirm the link, certain indicators may strongly suggest it.

## 4 Six signs that strengthens the case for the co-variation of somatic symptoms with emotional factors

**Figure 1:**
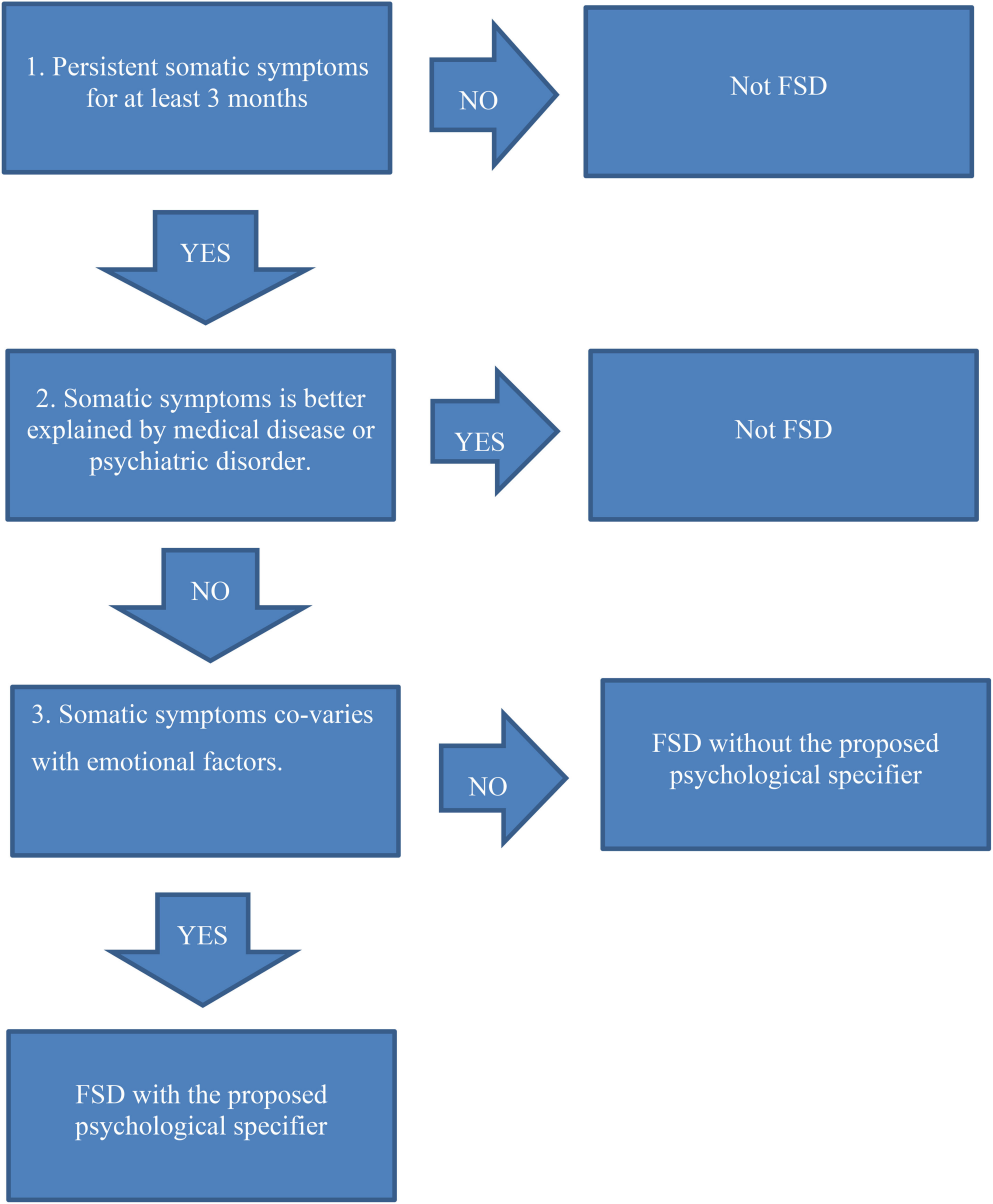
Characteristics of Functional Somatic Disorder with the new proposed psychological specifier.

While it is essential to begin with a null hypothesis during individual evaluations, it is equally important to identify potential indicators that could challenge this hypothesis. Below, I present six signs that may suggest a connection between somatic symptoms and emotional factors (see also **Table 3**). These signs are not definitive but serve as indicative markers (please see **Figure 1**).

**Table 3:** Six signs that strengthen the case that somatic symptoms co-vary with emotional factors.

1. **Temporal connection with emotional stress**:
○ Physical symptoms can emerge simultaneously or shortly after (up to a year) emotionally stressful life events. For example, a patient developing muscle weakness and weight loss following his wife’s critical illness suggests a possible association.
2. **Symptom fluctuation with emotional states**:
○ Symptoms that improve during relaxing situations (e.g., vacations) or worsen during stress indicate a potential emotional component. For example, a patient experiencing somatic symptoms when discussing his wife’s impending death suggests an emotional trigger for his symptoms.
3. **A plethora of somatic symptoms or changing nature of the symptoms**:
○ A plethora of physical symptoms or symptoms that frequently change or new symptoms that occur can indicate somatic symptoms connected to emotional factors rather than a fixed medical condition. This is supported by research showing that multiple unexplained symptoms are rarely due to an undiagnosed disease.
4. **Presence of “rigid beliefs” or defenses**:
○ Catastrophizing or rigid, fear-related thoughts are common among patients with functional symptoms. These thoughts can exacerbate pain and other symptoms, suggesting a psychological influence. For instance, a patient convinced he will end up in a wheelchair despite no medical evidence displays a rigid belief impacting his symptoms.
5. **Meaningful symptoms with emotional messages**:
○ Symptoms that seem to carry a personal or symbolic meaning might indicate an underlying emotional conflict. For example, exhausting fatigue may express unmet emotional needs or unresolved psychological conflicts, acting as a message from the body.
6. **Emotional quality in patient interaction**:
○ Health care professionals often develop a sense of whether somatic symptoms co-vary with emotional factors based on the quality of emotional interaction with the patient. This implicit knowledge can be used as a valuable indicator when assessing the association of emotional factors with physical symptoms.

### 4.1 Did the somatic symptoms occur simultaneously with or after a delay (up to one year) following emotionally stressful life events?

Psychological stressors often precede the development of functional symptoms, and stressful life events have been demonstrated to be risk factors for somatic symptom development in both crosssectional and longitudinal studies (26–31).

For instance, Lars, aged 73, was referred to me as a psychologist by his family physician. Lars revealed that he had experienced anxiety for most of his adult life, with his wife playing a crucial role in managing it. She helped him conceal his anxiety from others and devised alternative methods of transportation for him when he was unwilling to use the subway or train. A few months back, his wife was near death from heart disease, and Lars developed muscle weakness, reporting, “my legs don’t carry me anymore”. He also had difficulty eating and lost considerable weight. Despite numerous medical examinations, no medical explanation for his symptoms could be found. Lars denied being depressed and did not appear clinically depressed; he even smiled and shared a dry joke. The emergence of Lars’s physical symptoms coinciding with his wife’s terminal illness suggests that his symptoms may co-vary with emotional factors. While this is not a definitive sign, it is a plausible indicator.

The temporal connection between emotional stressors and physical symptoms merits a more nuanced discussion. Although psychological stressors often precede the development of functional symptoms, this is not always straightforward. In her book *The Thinking Body*, Irene Matthis uses Freud’s concept of “nachdräglichkeit” (deferred action) as pivotal in understanding somatic symptom development (32). Essentially, this concept implies that earlier experiences or events may gain their full meaning and impact only at a later time. Matthis examines cases from Freud’s Studies in Hysteria, such as the story of Katharina. During a walk in the Alpine landscape, Katharina, aware of Freud’s expertise as a neurologist, approached him with physical symptoms (chest pain, blurry vision, etc.) and sought his help. Through their discussion, Katharina revealed that she had witnessed sexual intercourse, which she initially believed was the cause of her symptoms. However, it emerged that this episode masked an earlier trauma: Katharina was sexually abused at the age of 11. As a child, she did not understand the significance of this abuse, and it only became meaningful later in adulthood when she encountered something that triggered her recollection of the trauma. Thus, now 19, Katharina developed symptoms several years after the triggering event when she comprehended the meaning of her experiences. Freud’s concept of nachdräglichkeit complicates the straightforward temporal connection between emotional factors and physical symptoms, but as a rule of thumb, the temporal connection of stressful life events and the development of somatic symptoms could be one sign of an important association.

### 4.2 Do the physical symptoms change in response to psychological or emotional factors?

If physical symptoms disappear during a vacation or when a person retreats to the countryside, or if they worsen during conflicts or before certain activities, this may indicate a relation between emotional factors and somatic symptoms (33). Unlike medical conditions, which usually persist regardless of a change in environment or emotional state, functional symptoms are heavily influenced by these factors (34).

For instance, in a conversation with Lars, I asked him to describe the moment he realized his wife could die from her heart disease. He struggled to articulate his feelings, his words faltering. He described it as if he were facing an abyss alone, overwhelmed by his anguish. When discussing the impending loss, Lars felt an overwhelming weakness in his legs, similar to the symptoms for which he sought treatment. This reaction suggests that his symptoms may be functional, as they are triggered by emotionally charged situations. While this is not a definitive sign, a recurring pattern would strongly indicate that his somatic symptoms co-vary with emotional factors. Further in treatment, Lars was not only able to express fear and anxiety but also emotions of anger and sadness for his wife “leaving him”. This led to a reduction of his somatic symptoms. This case parallels research that has shown that successful psychodynamic treatment for panic disorder is driven by, or probably causally related to, an increased capacity to express emotions (35). For an in-depth description of how this sign can be assessed, I refer the reader to Abbass (2005) (23).

### 4.3 Are there a multitude of physical symptoms, or do they change character or do new symptoms keep emerging?

This sign can be complex. While physical symptoms of medical conditions can vary and new symptoms may appear, most diseases tend to produce specific, primary symptoms rather than a broad spectrum. As Stefan Risberg, a senior family doctor, states: ”Most diseases produce distinct symptoms that are dominant, not a myriad of symptoms. The more varied the symptoms, the less likely it indicates an organic disease.“ (S. Risberg, personal communication, July 16 2024). Research supports this idea. A study investigating patients with multiple unexplained physical symptoms found that it is very uncommon for a physical disease to be overlooked in these cases, and it happened in only 2 of 2444 admissions (36).

More specifically, if a patient experiences, for example, pain that shifts from the arms to the neck and later to the head, this phenomenon, known as “symptom migration,” may indicate that emotional factors influence the somatic symptoms (37). In contrast, symptoms of physical illnesses typically remain localized. In the case of Lars, he described not only muscle weakness in the legs but also a plethora of other symptoms that emerged and then retracted: one week he had pain in the arms, the next week he had dry mouth and headaches.

### 4.4 Are defenses present?

Defenses can manifest in various forms. Catastrophizing, for instance, has been extensively studied in populations with persistent pain and fatigue. Catastrophizing involves pervasive “what if” thoughts, such as, “What if something serious is wrong?” and attention towards symptoms.

Catastrophizing is a potent psychological factor that can intensify the symptom experience, increase disability, and negatively impact treatment effectiveness (38, 39). Moreover, it aligns well with one of the B criteria specified in DSM-5 for SDD or BDD and is also listed as an important specifier from the EURONET-SOMA network.

In psychodynamically informed treatments, catastrophizing and fear-related thoughts are seen as factors that can exacerbate bodily symptoms. However, contrary to some psychological models, psychodynamically informed treatment’s view fear-related thoughts as defenses. These thoughts are believed to mask underlying emotions and unmet needs. To return to Lars, he had catastrophizing thoughts about not finding what was wrong with him. He was worried that if the doctor did not soon discover the underlying cause for his symptoms (remember that he had already undergone numerous medical investigations), he would get worse and become bedridden and need to be spoon-fed. This association of spoon-feeding revealed a memory where Lars, as a young boy, had been forcibly spoon-fed medicine by his father. Although the father probably had good intentions, this was very upsetting to Lars. He harbored angry feelings towards his father but at the same time felt guilt for these feelings. When Lars’s complex feelings (not only anger and guilt but love) towards his father were addressed and subsided, Lars became less anxious and less prone to catastrophizing. In other words, Lars’s defense—catastrophizing—needed to be addressed.

Moreover, from a psychodynamically informed standpoint, an absence of catastrophizing or anxietyladen thoughts and feelings should not necessarily rule out this criterion for SSD or the specifier in FSD. An unusual variant of catastrophic thinking is presenting fears as facts. For instance, Anders, a 19-year-old patient, had undergone three MRIs for his knee without any findings. He expressed his pain in a dispassionate manner, saying, “I will end up in a wheelchair”, without any emotional distress. His neutral tone was surprising, and his further response, “That’s how it is,” was emotionally muted. This detachment resembles what is sometimes referred to in psychodynamic literature as “le pense opératoire”, where patients appear unconcerned about serious symptoms, or the defense “isolation of affect”. In other words, it is possible that Anders use a defense which lessens the emotional impact of his pain, making the straightforward connection of somatic symptoms and emotional more difficult. Of course, there are numerous defenses to consider in investigating this sign.

### 4.5 Can the physical symptoms be meaningful and convey a message?

In psychodynamic theory, bodily symptoms are sometimes viewed as carriers of unconscious meaning (40). For instance, pain or other physical symptoms might not merely be indicators of an illness but could serve as messages or symbols. Psychodynamic theory often suggests that these meanings are highly personal rather than universal. To return to Lars, he described it as if he were facing an abyss alone when dreading the death of his spouse. A tentative interpretation of this is that you cannot walk over an abyss; there simply is no solid ground, and therefore Lars’s legs “could not carry [him] anymore.”

Another not uncommon example of symptoms as messages is found in patients with burnout/exhaustion syndrome. Many report that “The body said no!” (41). These accounts often reveal a history of ignoring early warning signs, struggling to set boundaries, or having difficulty saying no. Eventually, the body enforces these limits. On one level, the body can be seen as conveying a message. For patients with exhaustion syndrome, underlying desires to be cared for might surface. These individuals often prioritize others’ needs over their own but may have an underlying longing for support (42). In psychodynamic literature, the term counterdependency is sometimes used (43). Lawrence Blum elaborates on this concept: ”People with [FSD] often have conflicts where dependent wishes (e.g., to be cared for) are denied (typically expressed through a counterdependent, ‘I don’t need help’ stance). These unmet wishes are then expressed through physical symptoms.“ (L. Blum, personal communication, November 28 2020). Thus, physical symptoms such as fatigue may not only represent physiological issues but also reflect underlying emotional factors. Identifying such patterns can suggest that somatic symptoms are associated with emotional factors.

### 4.6 Is there an emotional quality in the encounter that suggests the somatic symptoms are connected to emotional factors?

This aspect is particularly challenging, as it relies heavily on the subjective impressions of the assessor. Despite its difficulty, it can be crucial. Over time, health care professionals develop a form of implicit knowledge—a type of intuitive understanding based on extensive experience (44).

According to psychodynamic theory, understanding one’s own countertransference (emotional reactions in the health provider towards the patient) is important. In patient encounters, there may be an emotional quality that indicates that somatic symptoms co-vary with emotional factors. This quality is subtle and difficult to describe but can involve a sense of emotional detachment or muted interaction. It is not necessarily about the absence of emotional words or exchanges but rather a feeling of engaging through a barrier, like touching something using thin plastic gloves. While standardized methods are crucial for identifying signs of the association of somatic symptoms and emotional factors, implicit knowledge and judgments from this knowledge based on countertransference also play an important role.

Taken together, these six signs may help pinpoint patients who have an association of emotional factors and somatic symptoms. Assessing these signs will incorporate evaluating signs both for and against the connection between physical and emotional factors. No single indicator is definitive, but patterns can provide strong indications.

## 5 Discussion

In this article, I propose that the current DSM-5 criteria for Somatic Symptom Disorder (SSD), the ICD-11 criteria for Bodily Distress Disorder (BDD), and the Functional Somatic Disorder (FSD) psychological specifier could be expanded to include an assessment of whether emotional factors covary with somatic symptoms. I have highlighted the importance of a collaborative approach between the patient and investigator to achieve a more comprehensive understanding and suggested six signs to consider when evaluating the interplay between emotional factors and physical symptoms in patients with FSD. Incorporating this specifier is crucial not only for determining an association between emotional factors and somatic symptoms but also for assessing the appropriateness of affect focused psychodynamic treatment, which is an evidence-based treatment for FSD. Furthermore, it offers valuable insights into the broader context of symptom management and treatment planning, and the six signs are quite accessible to use for a range of health-care professionals.

The recent advocacy for the term “Functional Somatic Disorder” by the EURONET-SOMA group represents a step forward in framing these conditions within a modern diagnostic framework. The addition of a new specific psychological specifier or the widening of the criteria for SSD and BDD, as previously suggested (45), could enhance the FSD framework by acknowledging the role of emotional factors in the manifestation and perpetuation of physical symptoms. Previous research has shown that emotional factors (such as emotional regulation) are understudied in the context of SSD/FSD (46) and that the emphasis has been placed on cognitive-behavioral signs as the basis for SSD (47). However, the psychodynamically informed approach proposes that emotional factors such as unresolved grief, trauma, or unmet needs may exacerbate or even cause somatic symptoms (48). This perspective aligns with contemporary psychodynamic theories, which posit that emotional distress can manifest as physical symptoms due to maladaptive emotional processing or unresolved conflicts (49). By incorporating emotional factors into the diagnostic criteria, clinicians can more accurately identify patients whose symptoms may be influenced by psychological factors. This can lead to more targeted and effective treatment strategies, potentially reducing the reliance on purely medical or symptomatic approaches.

Given the demonstrated effectiveness of EAET and ISTDP in targeting emotional processing deficits in FSD, incorporating emotional factors into diagnostic frameworks could improve treatment selection. Moreover, understanding that somatic symptoms may carry personal meaning or reflect deeper emotional issues enables clinicians to provide personalized care (50). This approach respects the individuality of each patient’s experience and seeks to address the unique emotional and psychological factors contributing to their symptoms.

However, there are several challenges with incorporating a new criterion for SSD/BDD or a new specifier for FSD. One of the primary challenges in incorporating emotional factors into the FSD specifier or a criterion for SSD/BDD is the inherent subjectivity involved in assessing these factors. Emotional responses and their influence on physical symptoms are often difficult to quantify and may vary significantly between individuals, but previous research using micro-longitudinal design with ecological momentary assessment has shown that it is possible (51). I believe that the six signs or questions are quite accessible to a range of healthcare professionals when assessing if emotional factors co-vary with somatic symptoms, but their validity and reliability have not yet been tested in large-scale clinical studies. Of course, ensuring that the assessment of emotional factors is both reliable and valid requires careful consideration and potentially the development of standardized methods for evaluation. Further research is needed to validate the proposed specifier and its impact on treatment outcomes. This could be achieved through reliability studies assessing interrater agreement or by correlating the specifier of emotional factors with other measures, such as deficits in emotional processing. Moreover, longitudinal studies may also be valuable in determining whether this specifier predict treatment response or long-term symptom trajectories, thereby further establishing its clinical utility.

Another challenge is establishing rapport with patients who often have low epistemic trust (52). The collaborative approach proposed for investigating the connection between emotional and physical symptoms necessitates clear communication and mutual consent between the patient and clinician. Patients must be willing to explore the potential emotional roots of their symptoms, which may not always align with their expectations or treatment preferences. Ensuring that patients are fully informed and agree to this exploration is essential for the process to be effective.

Thirdly, incorporating emotional factors into the diagnostic criteria raises considerations regarding the potential for overemphasis on specific psychological factors. It is important to balance the focus on emotional factors with a comprehensive evaluation of all relevant medical and psychiatric conditions to avoid misdiagnosis or stigmatization. The new criteria should not suggest that all persistent physical symptoms and syndromes are psychiatric, nor that there are no biological abnormalities. For instance, in patients with hemiplegia where brain damage has been ruled out with great certainty, there are still well-documented changes in the way the brain works and functions (53). The difference is that for a stroke patient, the brain injury cannot be healed. However, in the case of functional paralysis, where there is a pattern of symptoms co-varying with emotional factors, recovery is possible, although the work to get there is just as hard and arduous. As for Lars, he has regained weight, is able to walk again, and has continually challenged his anxiety by traveling to new places he did not dare visit previously, being alone without his wife (which he dreaded), and acknowledging his anxiety to friends (for which he felt ashamed).

Another important point is that patients are complex. For instance, about 20% of individuals with non-epileptic seizures also have epilepsy (54), non-cardiac chest pain frequently occurs after a myocardial infarction (55), and dysfunctional breathing is often observed in those with asthma (56). The work of delineating which somatic symptoms co-vary with emotional factors can be difficult and should always include collaboration with numerous health care professionals (57).

In conclusion, the proposal to include a psychodynamically informed specifier in the classification of FSD or a new criterion in SSD/BDD represents a significant advancement in understanding and treating patients with persistent physical symptoms. By recognizing the role of emotional factors, clinicians can provide more nuanced and effective care, addressing both the physical and psychological dimensions of the disorder. However, successful implementation requires careful consideration of subjectivity, patient collaboration and empirical validation. As research and clinical practice continue to evolve, the integration of emotional factors into the FSD framework holds promise for improving diagnostic accuracy and therapeutic outcomes, ultimately leading to better patient care and well-being.

## References

[1] Henningsen P Hausteiner-Wiehle C Häuser W. Migraine in the context of chronic primary pain, chronic overlapping pain disorders, and functional somatic disorders: A narrative review. Headache J Head Face Pain. (2022) 62:1272–80. doi: 10.1111/head.14419 36373821

[2] Wessely S Nimnuan C Sharpe M. Functional somatic syndromes: One or many? Lancet. (1999) 354:936–9. doi: 10.1016/S0140-6736(98)08320-2 10489969

[3] Yunus MB. Fibromyalgia and overlapping disorders: the unifying concept of central sensitivity syndromes. Semin Arthritis Rheum. (2007) 36:339–56. doi: 10.1016/j.semarthrit.2006.12.009 17350675

[4] Löwe B Toussaint A Rosmalen JGM Huang W-L Burton C Weigel A. Persistent physical symptoms: definition, genesis, and management. Lancet. (2024) 403:2649–62. doi: 10.1016/S0140-6736(24)00623-8 38879263

[5] American Psychiatric Association. Diagnostic and statistical manual of mental disorders. 5th ed. American Psychiatric Publishing (2013).

[6] World Health Organization. International Classification of Diseases, 11th Revision (ICD-11). (2019).

[7] on behalf of the EURONET-SOMA Group Burton C Fink P Henningsen P Löwe B Rief W. Functional somatic disorders: discussion paper for a new common classification for research and clinical use. BMC Med. (2020) 18:34. doi: 10.1186/s12916-020-1505-4 32122350 PMC7052963

[8] Löwe B Levenson J Depping M Hüsing P Kohlmann S Lehmann M. Somatic symptom disorder: a scoping review on the empirical evidence of a new diagnosis. Psychol Med. (2022) 52:632–48. doi: 10.1017/S0033291721004177 PMC8961337 34776017

[9] Abbass A Town J Holmes H Luyten P Cooper A Russell L. Short-term psychodynamic psychotherapy for functional somatic disorders: A meta-analysis of randomized controlled trials. Psychother Psychosom. (2020) 89:363–70. doi: 10.1159/000507738 32428905

[10] Abbass A Lumley MA Town J Holmes H Luyten P Cooper A. Short-term psychodynamic psychotherapy for functional somatic disorders: A systematic review and metaanalysis of within-treatment effects. J Psychosom Res. (2021) 145:110473. doi: 10.1016/j.jpsychores.2021.110473 33814192

[11] Lumley MA Schubiner H Lockhart NA Kidwell KM Harte SE Clauw DJ. Emotional awareness and expression therapy, cognitive behavioral therapy, and education for fibromyalgia: a cluster-randomized controlled trial. Pain. (2017) 158:2354–63. doi: 10.1097/j.pain.0000000000001036 PMC5680092 28796118

[12] Yarns BC Lumley MA Cassidy JT Steers WN Osato S Schubiner H. Emotional awareness and expression therapy achieves greater pain reduction than cognitive behavioral therapy in older adults with chronic musculoskeletal pain: A preliminary randomized comparison trial. Pain Med. (2020) 21:2811–22. doi: 10.1093/pm/pnaa145 32451528

[13] Yarns BC Jackson NJ Alas A Melrose RJ Lumley MA Sultzer DL. Emotional awareness and expression therapy vs cognitive behavioral therapy for chronic pain in older veterans: A randomized clinical trial. JAMA Netw Open. (2024) 7:e2415842. doi: 10.1001/jamanetworkopen.2024.15842 38869899 PMC11177167

[14] Leichsenring F Abbass A Heim N Keefe JR Kisely S Luyten P. The status of psychodynamic psychotherapy as an empirically supported treatment for common mental disorders – an umbrella review based on updated criteria. World Psychiatry. (2023) 22:286–304. doi: 10.1002/wps.21104 37159376 PMC10168167

[15] Lumley MA Schubiner H. Emotional awareness and expression therapy for chronic pain: rationale, principles and techniques, evidence, and critical review. Curr Rheumatol Rep. (2019) 21:30. doi: 10.1007/s11926-019-0829-6 31123837 PMC7309024

[16] Okur Güney ZE Sattel H Witthöft M Henningsen P. Emotion regulation in patients with somatic symptom and related disorders: A systematic review. PloS One. (2019) 14:e0217277. doi: 10.1371/journal.pone.0217277 31173599 PMC6555516

[17] Frisch S Walter S Rebhann V Gruss S Geisel D Bär K-J. Unconscious activation of negative emotional memories increases pain unpleasantness. Psychosom Med. (2024) 86:580–90. doi: 10.1097/psy.0000000000001315 38666650

[18] Eisenberger NI Lieberman MD. Why rejection hurts: a common neural alarm system for physical and social pain. Trends Cognit Sci. (2004) 8:294–300. doi: 10.1016/j.tics.2004.05.010 15242688

[19] Pinto AM Geenen R Wager TD Lumley MA Häuser W Kosek E. Emotion regulation and the salience network: a hypothetical integrative model of fibromyalgia. Nat Rev Rheumatol. (2023) 19:44–60. doi: 10.1038/s41584-022-00873-6 36471023

[20] Büchel C Geuter S Sprenger C Eippert F. Placebo analgesia: A predictive coding perspective. Neuron. (2014) 81:1223–39. doi: 10.1016/j.neuron.2014.02.042 24656247

[21] Maroti D Ljótsson B Lumley MA Schubiner H Hallberg H Olsson P-Å. Emotional processing and its association to somatic symptom change in emotional awareness and expression therapy for somatic symptom disorder: A preliminary mediation investigation. Front Psychol. (2021) 12:712518. doi: 10.3389/fpsyg.2021.712518 34690868 PMC8528965

[22] Maroti D Lumley MA Schubiner H Lilliengren P Bileviciute-Ljungar I Ljótsson B. Internet-based emotional awareness and expression therapy for somatic symptom disorder: A randomized controlled trial. J Psychosom Res. (2022) 163:111068. doi: 10.1016/j.jpsychores.2022.111068 36327532

[23] Abbass A. Somatization: Diagnosing it sooner through emotion-focused interviewing. Psychotherapy: Theory, Research, Practice, Training. (2005) 42(4):513–17 doi: 10.1037/0033-3204.42.4.513 15755376

[24] Husain M Chalder T. Medically unexplained symptoms: assessment and management. Clin Med. (2021) 21:13–8. doi: 10.7861/clinmed.2020-0947 PMC7850206 33479063

[25] Ventres W. PRESSS: A new patient-centered name for an old problem. J Am Board Fam Med. (2021) 34:1030–2. doi: 10.3122/jabfm.2021.05.200647 34535530

[26] Puig J Englund MM Simpson JA Collins WA. Predicting adult physical illness from infant attachment: A prospective longitudinal study. Health Psychol. (2013) 32:409–17. doi: 10.1037/a0028889 PMC3480992 22823067

[27] Felitti VJ Anda RF Nordenberg D Williamson DF Spitz AM Edwards V. Relationship of childhood abuse and household dysfunction to many of the leading causes of death in adults. Am J Prev Med. (1998) 14:245–58. doi: 10.1016/S0749-3797(98)00017-8 9635069

[28] Afari N Ahumada SM Wright LJ Mostoufi S Golnari G Reis V. Psychological trauma and functional somatic syndromes: A systematic review and meta-analysis. Psychosom Med. (2014) 76:2–11. doi: 10.1097/PSY.0000000000000010 24336429 PMC3894419

[29] Beesley H Rhodes J Salmon P. Anger and childhood sexual abuse are independently associated with irritable bowel syndrome. Br J Health Psychol. (2010) 15:389–99. doi: 10.1348/135910709X466496 19691916

[30] Jones GT Power C Macfarlane GJ. Adverse events in childhood and chronic widespread pain in adult life: Results from the 1958 British Birth Cohort Study. Pain. (2009) 143:92–6. doi: 10.1016/j.pain.2009.02.003 19304391

[31] Kivimaki M Leinoarjas P Virtanen M Elovainio M Keltikangasjarvinen L Puttonen S. Work stress and incidence of newly diagnosed fibromyalgiaProspective cohort study. J Psychosom Res. (2004) 57:417–22. doi: 10.1016/S0022-3999(03)00620-2 15581643

[32] Matthis I. Den tänkande kroppen. Natur och Kultur (1997).

[33] Abbass A Schubiner H. Hidden from view: A clinician’s guide psychophysiological disorders. LLC: Psychophysiological press (2018).

[34] Hausteiner-Wiehle C Henningsen P. Nociplastic pain is functional pain. Lancet. (2022) 399:1603–4. doi: 10.1016/S0140-6736(21)02500-9 35461549

[35] Keefe JR Huque ZM DeRubeis RJ Barber JP Milrod BL Chambless DL. In-session emotional expression predicts symptomatic and panic-specific reflective functioning improvements in panic-focused psychodynamic psychotherapy. Psychotherapy. (2019) 56:514–25. doi: 10.1037/pst0000215 30869969 PMC6745012

[36] Fink P. Admission patterns of persistent somatization patients. Gen Hosp Psychiatry. (1993) 15:211–8. doi: 10.1016/0163-8343(93)90035-M 8344510

[37] Schubiner H Betzold M. Unlearn your pain. Mind Body publishing (2010).

[38] Lukkahatai N Saligan LN. Association of catastrophizing and fatigue: A systematic review. J Psychosom Res. (2013) 74:100–9. doi: 10.1016/j.jpsychores.2012.11.006 PMC3553502 23332523

[39] Quartana PJ Campbell CM Edwards RR. Pain catastrophizing: a critical review. Expert Rev Neurother. (2009) 9:745–58. doi: 10.1586/ern.09.34 PMC2696024 19402782

[40] Maroti D. Smärtans budskap. Borell (2022).

[41] Mate G. When the body says no - The Cost of Hidden Stress. John Wiley & sons, Inc (2003).

[42] Guzzi DA. Redefining burnout: exploring common conceptualizations and the neurophysiology of chronic stress to establish an integrated allostatic model. Dissertations. (2019) 374. Available online at: https://digitalcommons.nl.edu/diss/374.

[43] Blum LD Horenstein A Carper MM Stange JP Cohen JN Doyle A. A new instrument to assess counterdependency, evaluated in the context of postpartum depression. Psychoanal Psychol. (2021) 38:49–57. doi: 10.1037/pap0000317

[44] Schacter DL. Implicit knowledge: new perspectives on unconscious processes. Proc Natl Acad Sci. (1992) 89:11113–7. doi: 10.1073/pnas.89.23.11113 PMC50499 1454787

[45] Mewes R. Recent developments on psychological factors in medically unexplained symptoms and somatoform disorders. Front Public Health. (2022) 10:1033203. doi: 10.3389/fpubh.2022.1033203 36408051 PMC9672811

[46] Smakowski A Hüsing P Völcker S Löwe B Rosmalen JGM Shedden-Mora M. Psychological risk factors of somatic symptom disorder: A systematic review and meta-analysis of cross-sectional and longitudinal studies. J Psychosom Res. (2024) 181:111608. doi: 10.1016/j.jpsychores.2024.111608 38365462

[47] Maggio J Alluri PR Paredes-Echeverri S Larson AG Sojka P Price BH. Briquet syndrome revisited: implications for functional neurological disorder. Brain Commun. (2020) 2:fcaa156. doi: 10.1093/braincomms/fcaa156 33426523 PMC7784044

[48] Abbass A. Reaching through resistance. Advanced psychotherapy techniques. Seaven Leaves Press (2015).

[49] Scamvougeras A Castle D. Functional neurological disorders: challenging the mainstream agnostic causative position. Can J Psychiatry. (2024) 69:487–92. doi: 10.1177/07067437241245957 PMC11168345 38584382

[50] Srinivas SK Anant S. Her body grieves her unsung pain: traumatic life experiences of women living with medically unexplained pain. Ind J Clin Psych. (2023) 49:01–6.

[51] Klaus K Fischer S Doerr JM Nater UM Mewes R. Classifying fibromyalgia syndrome as a mental disorder?—An ambulatory assessment study. Int J Behav Med. (2017) 24:230–8. doi: 10.1007/s12529-016-9603-6 27757841

[52] Luyten P Fonagy P. Psychodynamic psychotherapy for patients with functional somatic disorders and the road to recovery. Am J Psychother. (2020) 73:125–30. doi: 10.1176/appi.psychotherapy.20200010 33203227

[53] Benussi A Premi E Cantoni V Compostella S Magni E Gilberti N. Cortical inhibitory imbalance in functional paralysis. Front Hum Neurosci. (2020) 14:153. doi: 10.3389/fnhum.2020.00153 32457588 PMC7220997

[54] Mellers JDC. The approach to patients with “non-epileptic seizures. ” Postgrad Med J. (2005) 81:498–504. doi: 10.1136/pgmj.2004.029785 16085740 PMC1743326

[55] Qintar M Spertus JA Tang Y Buchanan DM Chan PS Amin AP. Noncardiac chest pain after acute myocardial infarction: Frequency and association with health status outcomes. Am Heart J. (2017) 186:1–11. doi: 10.1016/j.ahj.2017.01.001 28454822 PMC5575910

[56] Boulding R Stacey R Niven R Fowler SJ. Dysfunctional breathing: a review of the literature and proposal for classification. Eur Respir Rev. (2016) 25:287–94. doi: 10.1183/16000617.0088-2015 PMC9487208 27581828

[57] Czachowski S. Functional disorders – new proposals for definition, psychosomatics, somatization. Psychiatr Pol. (2023) 57:421–30. doi: 10.12740/PP/OnlineFirst/141960 37350707

